# Combining Machine Learning With Real-World Data to Identify Gaps in Clinical Practice Guidelines: Feasibility Study Using the Prospective German Stroke Registry and the National Acute Ischemic Stroke Guidelines

**DOI:** 10.2196/69282

**Published:** 2025-07-11

**Authors:** Sandrine Müller, Susanne Diekmann, Markus Wenzel, Horst Karl Hahn, Johannes Tuennerhoff, Ulrike Ernemann, Florian Hennersdorf, German Stroke Registry Investigators, Arno Reich, Max Westphal, Sven Poli

**Affiliations:** 1 Fraunhofer Institute for Digital Medicine, Bremen, Germany; 2 Constructor University, Bremen, Germany; 3 Department of Mathematics and Computer Science, University of Bremen, Bremen, Germany; 4 Department of Neurology & Stroke, University of Tübingen, Hoppe-Seyler-Str. 3, Tübingen, 72076, Germany, 49 1724682284; 5 Hertie Institute for Clinical Brain Research, Tübingen, Germany; 6 Department of Diagnostic and Interventional Neuroradiology, University of Tübingen, Tübingen, Germany

**Keywords:** clinical practice guidelines, real-world data, machine learning, acute ischemic stroke, health care improvement, guideline adherence

## Abstract

**Background:**

Clinical practice guidelines (CPGs) serve as essential tools for guiding clinicians in providing appropriate patient care. However, clinical practice does not always reflect CPGs. This is particularly critical in acute diseases requiring immediate treatment, such as acute ischemic stroke, one of the leading causes of morbidity and mortality worldwide. Adherence to CPGs improves patient outcomes, yet guidelines may not address all patient scenarios, resulting in variability in treatment decisions. Identifying such gaps would augment CPGs but is challenging when using traditional methods.

**Objective:**

This study aims to leverage real-world data coupled with machine learning (ML) techniques to systematically identify and quantify gaps in German thrombolysis-in-stroke guidelines.

**Methods:**

We analyzed observational data from the German Stroke Registry – Endovascular Treatment (GSR-ET), a prospective national registry involving 18,069 patients from 25 stroke centers in whom endovascular treatment of a large vessel occlusion was attempted between 2015 and 2023. Key variables included demographic, clinical and imaging information, treatment details, and outcomes. A random forest model was used to predict intravenous thrombolysis treatment decisions based on three different sets of features: (1) guideline-recommended features, (2) clinician-selected features, and (3) features as documented in the GSR-ET before thrombolytic treatment. Feature importance scores, permutation importance, and Shapley Additive Explanations values were used, with clinician guidance, to interpret the model and identify key factors associated with guideline deviations and independent clinician judgments.

**Results:**

Of all GSR-ET patients, 13,440 (74.4%) were analyzed after excluding those with incomplete or implausible data. The random forest model’s performance, measured by area under the receiver operating characteristics curve, was 0.71 (95% CI 0.68‐0.73), 0.74 (95% CI 0.73‐0.75), and 0.77 (95% CI 0.76‐0.78) for the guideline-recommended, clinician-selected, and GSR-ET feature sets, respectively. Across all sets, time from symptom onset to admission was the most important predictor of thrombolysis treatment decisions. Age, which according to the German guidelines is not to be considered for thrombolysis administration, emerged as a significant predictor in the GSR-ET feature set, suggesting a potential gap between guidelines and clinical practice.

**Conclusions:**

In our study, we introduce an innovative approach that combines real-world data with ML techniques to identify discrepancies between CPGs and actual clinical decision-making. Using intravenous thrombolysis in large vessel occlusion stroke as a model, our findings suggest that treatment decisions may be influenced by factors not explicitly included in the current German guideline, such as patient age and pre-stroke functional status. This approach may help uncover clinically relevant variables for potential inclusion in future guideline refinements.

## Introduction

### Background

Ischemic stroke is a major global health concern being one of the leading causes of death and long-term disability worldwide, with more than 6 million deaths reported in 2019 [1]. Effective management of ischemic stroke is critical for lowering morbidity and mortality [2]. Clinical practice guidelines (CPGs), developed by expert panels and health care organizations, are part of an evidence-based practice toolkit designed to standardize medical treatment and ensure that patients receive the best possible care. The acute ischemic stroke guidelines encompass recommendations for pre- and in-hospital care, early diagnosis, timely administration of intravenous thrombolysis, and the implementation of endovascular thrombectomy as needed [3-5].

Despite the availability of these guidelines and the effort made to keep them current, adherence in clinical practice varies greatly [6,7]. Physicians may rely on independent clinical judgment in the absence of specific recommendations or intentionally deviate from guidelines due to several reasons, including addressing individual patient needs [8-10]. Such situations expose potential gaps in the guidelines, indicating areas where further guidance could improve standardization and patient care. In our study, a gap in CPGs refers to a discrepancy between guideline recommendations and the decisions observed in real-world clinical scenarios. According to research, deviations from recommended practices and the need for clinical discretion when no guidance is provided can result in suboptimal outcomes including increased rates of death and disability [11,12]. Currently employed traditional methods of detecting gaps in guidelines, such as comparing national and international guidelines and manual chart reviews, are labor-intensive, time-consuming, and susceptible to human error [13-17].

Recent research has shown that artificial intelligence and machine learning (ML) are effective in various health care applications, including predicting patient outcomes, identifying risk factors, and assisting clinicians with decision-making [18-21]. Another promising way to enhance patient outcomes in health care is the usage of real-world data, which includes routinely collected data (eg, electronic health records and registry data) to optimize treatment protocols based on real-world evidence [22]. There has been limited research within the realm of CPGs aimed at generating or enhancing health care guidelines by leveraging ML and real-world data [23-27]. To the best of our knowledge, the application of ML algorithms combined with real-world data to identify gaps in CPGs remains unexplored. Analyzing routine data with ML algorithms, such as random forests, which can handle complex interactions between variables and provide insights into the relative importance of various factors influencing clinical decisions, can be pivotal in systematically identifying areas where clinical practice diverges from CPGs, including areas not covered by existing recommendations. This approach can pinpoint specific deficiencies in CPGs and highlight potential areas for improvement, thereby offering a valuable addition to the traditional methods used for identifying gaps in CPGs.

### Objective

To investigate whether intravenous thrombolysis treatment decisions follow the current German acute ischemic stroke guideline or are informed by additional features that are not part of the guideline recommendations. Specifically, we aim to:

1. Assess the performance of an ML model in predicting thrombolysis treatment when implemented on 3 different sets of features.
2. Identify key factors in thrombolysis administration that are currently not included in the guidelines.

## Methods

### Data Collection

The data used in this study were retrieved from the German Stroke Registry – Endovascular Treatment (GSR-ET) [28], a nationwide registry comprising pseudonymized, prospectively documented records of 18,069 patients who underwent endovascular stroke treatment as part of routine clinical care at 25 stroke centers across Germany between 2015 and 2023. For this study, we extracted patient-level data from structured fields in the GSR-ET, limited to variables available before the intravenous thrombolysis decision, thereby ensuring clinical relevance at the time of decision-making.

The dataset included the demographic age and sex, pre-stroke functional status, as measured by the modified Rankin Scale (mRS); relevant comorbidities included previous stroke, diabetes mellitus, dyslipidemia, arterial hypertension, and atrial fibrillation, as well as smoking status and antiplatelet or anticoagulation therapy at stroke onset. Living status before stroke was categorized as living at home, in a nursing home, or receiving nursing services at home.

At the time of admission, clinical variables included stroke severity, assessed using the National Institutes of Health Stroke Scale (NIHSS), and systolic and diastolic blood pressure. Radiological data included imaging modalities (ie, noncontrast enhanced computed tomography, CT angiography [CTA], computed tomography [CT] perfusion, magnetic resonance imaging, magnetic resonance angiography [MRA], magnetic resonance perfusion, and imaging-based findings such as the Alberta Stroke Program Early CT Score [ASPECTS]), indicating the extent of ischemic brain tissue damage; the Thrombolysis in Cerebral Infarction (TICI) score on CTA or MRA, indicating the degree of orthograde perfusion of the affected territory; the laterality (left, right, and bilateral) and location of the vessel occlusion (anterior or posterior circulation); the presence of tandem stenosis, and signs of ischemia outside the occluded vessel territory.

Process-related variables included the time from symptom onset (or last seen well) to hospital admission and whether the patient was primarily admitted to an interventional stroke center.

The primary outcome of interest in this study was whether a patient received intravenous thrombolysis before endovascular therapy.

### Data Cleaning and Preparation

**Figure 1.**
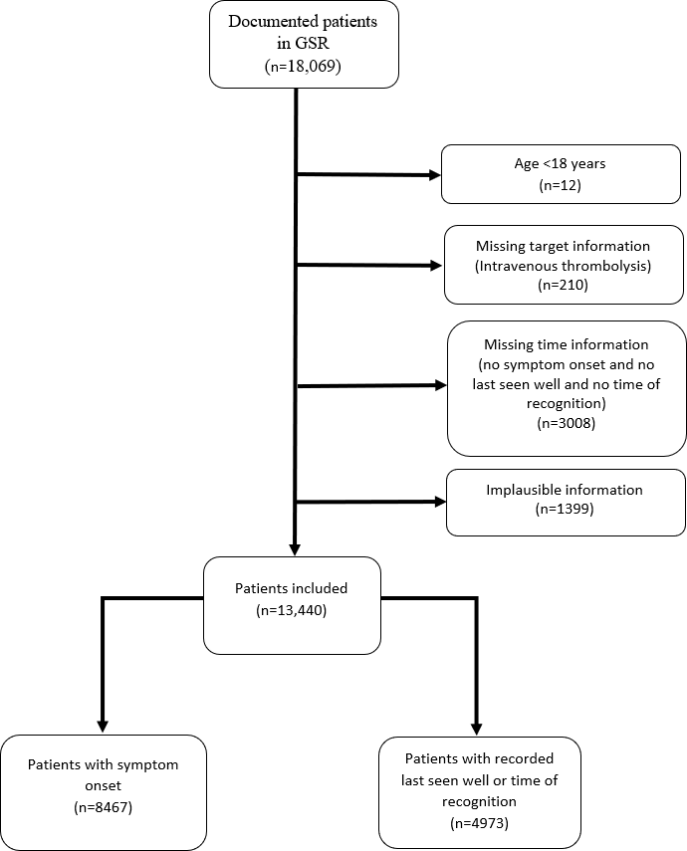
Flowchart illustrating patient selection. GSR: German Stroke Registry.

Before analysis, the data was cleaned, and patients were excluded based on prespecified exclusion criteria including incomplete or implausible data (see Figure 1 and Multimedia Appendix 1). To estimate missing values for age, admission blood pressure, scores on the NIHSS, and the pre-stroke mRS score, we employed the multiple imputation by chained equations technique, provided in the Python library [29]. To assess the robustness of our imputation strategy, we compared model performance by dropping missing values and using mean and k-nearest neighbor imputation strategies. To reduce the risk of multicollinearity, we performed correlation analysis using the Pearson method for numerical variables and Cramer V for categorical variables. In addition, the reported antithrombotic medication intake by patients was grouped into 2 categories: anticoagulants (apixaban, rivaroxaban, edoxaban, dabigatran, phenprocoumon, and heparins) and antiplatelets (aspirin, clopidogrel, and others). This approach helped manage correlation between variables, ensuring that each group represented a distinct set of clinical effects. Comorbidity features such as hypertension, diabetes mellitus, atrial fibrillation, and dyslipidemia were excluded from the analysis as we were not sure when exactly this information was documented (before or after the target feature). Occluded vessel features were grouped into 2 distinct groups: anterior and posterior circulation occlusion. Categorical and ordinal variables were transformed using one-hot encoding and ordinal encoding respectively to ensure compatibility with the ML model.

Out of the 206 available features, a total of 26 features all obtained before the target feature and relevant to thrombolysis administration were extracted from the GSR-ET. To measure the performance of our random forest model in predicting initiation of thrombolytic treatment, we created 3 distinct feature sets (see Multimedia Appendix 2 for more details) that we call the “Guideline”, the “Clinician”, and the “GSR-ET” feature sets and define as follows:

- Guideline feature set: this feature set includes the 11 features explicitly outlined in the German acute ischemic stroke guideline which are deemed relevant for intravenous thrombolysis decision-making. The inclusion of these features was based on their established clinical relevance in assessing treatment eligibility and aligning with evidence-based practice. The guideline feature set was chosen to directly reflect the guideline’s criteria, ensuring the model is evaluated against well-defined clinical standards.
- Clinician feature set: this feature set is derived from expert clinician judgment and includes 18 features identified by 2 independent stroke physicians (TJ and PS) as crucial for thrombolysis decision-making. Selection was based on their real-world experience and understanding of a broader range of patient factors that influence treatment decisions. While some of these features may not be explicitly outlined in formal guidelines, they are considered essential in clinical practice.

- GSR-ET feature set: this feature set includes all 26 features from the GSR-ET dataset that are documented before thrombolysis administration. Their inclusion ensures that the model captures a comprehensive view of the patient’s condition as recorded by clinicians in real-world settings, incorporating factors that may influence treatment decisions beyond what is specified by guidelines or expert judgment.

### Outcome of Interest

Treatment with intravenous thrombolysis was chosen as the outcome of interest for this study. Thrombolysis is a critical and time-sensitive treatment for acute ischemic stroke [30-36], and it offers a clear, binary outcome suitable for classification algorithms, making it an ideal target for ML models such as random forests. In addition, thrombolysis represents an important quality indicator of stroke care. Despite known contraindications and gaps in existing guidelines, clinicians rely on their independent judgment and often administer thrombolysis, because it, along with endovascular thrombectomy, represents the only effective treatment option in the acute phase of stroke. We consider predicting intravenous thrombolysis treatment valuable as it captures the initial and crucial acute stroke decision-making process.

### Machine Learning Model

To efficiently identify a suitable classification model for our analysis, we tested various ML algorithms using the lazypredict library [37], which quickly trains and compares the performance of many different classifiers on a given dataset.

Based on whether symptom onset was known or not, patients were divided into 2 subgroups for which we suspect possible different behavior concerning the target variable:

- Group A: Recorded symptom onset (n=8467, 62.9% patients).
- Group B: Recorded “last seen well” or “time of symptom recognition” (n=4973, 37.1% patients).

All subsequent steps of model development, training, and evaluation were conducted independently within each subgroup to ensure that analyses reflect their unique clinical and decision-making characteristics.

The dataset was split into training (80%) and validation (20%) sets for both subgroups to separate training and validation of the models and thereby detect overfitting and allow for a robust performance evaluation.

To optimize model performance, we used the nested cross-validation approach [38]. We performed an inner cross-validation using RandomizedSearchCV from the Python library scikit-learn on the training set to tune hyperparameters [39]. The hyperparameter search space included the number of estimators (n_estimators: 50‐500) and the maximum depth of trees (max-depth: 1‐20). A total of 5 hyperparameter configurations were randomly sampled, balancing exploration with computational efficiency. The model was evaluated across different combinations of features, and the best-performing configuration from the process was selected for final evaluation on the validation set.

### Model Performance Assessment and Interpretability

Our model was evaluated on the validation set, primarily using the area under the receiver operating characteristic curve (AUROC). To ensure robustness, maintain class proportions, and prevent overfitting, we performed an outer cross-validation using stratified k-fold cross-validation (with k=5) on the tuned model to derive AUROC results. This method divides the training set into 5 subsets (4 for training and 1 for validation) and ensures that each fold of the cross-validation maintains the same proportion of class labels, which is particularly important given the potential imbalance in the chosen treatment (intravenous thrombolysis). We further addressed class imbalance by applying the synthetic minority over-sampling technique within the cross-validation loop to oversample the minority class in the training data. Secondary performance measures included F_1_-score, accuracy, precision, and recall.

To assess the importance of each feature in their respective sets, in predicting intravenous thrombolysis treatment, we used a comprehensive analysis combining several methods. Initially, feature importance scores were extracted from the random forest model to provide a foundational understanding of each feature’s contribution. To capture each feature’s impact on model accuracy, permutation feature importance was applied. This loss-based approach quantifies feature importance by measuring the decrease in model performance when individual features are randomly permuted [40]. In addition, to ensure the robustness of the feature importance evaluations, we computed the confidence intervals for each feature within its respective feature set based on permutation importance results, applying the correlated t-test approach [41,42]. This method enabled us to visualize the variability and reliability of the feature importance scores. Finally, Shapley Additive Explanations (SHAP), a variance-based method, was also employed to examine the influence of each feature at the prediction level, offering deeper insights into feature contributions to both predictions (intravenous thrombolysis or no intravenous thrombolysis) [43]. Together, these methods enabled a thorough exploration of features that play an important role in the model’s predictions.

### Statistical Analyses

For categorical features, absolute and relative frequencies were computed. The interquartile ranges, minimum and maximum values of continuous features including NIHSS (ordinal), and pre-stroke mRS (ordinal) were also computed. The chi-square test was used to compare proportions between groups for categorical variables. For continuous and ordinal variables, the Wilcoxon rank-sum test was applied.

The descriptive comparison focused on the differences between patients who received intravenous thrombolysis and those who did not. These included demographic characteristics (eg, age, sex, and living status), comorbidities (eg atrial fibrillation, diabetes, and previous stroke), imaging modalities, occlusion sites, and pretreatment clinical status (eg NIHSS and pre-stroke mRS).

To quantify uncertainty in the AUROC estimates, 95% CIs were calculated using the correlated *t* test. All tests were 2-sided, and a *P* value of <.05 was considered statistically significant. Given the exploratory nature of the analysis, *P* values were not adjusted for multiple testing. Statistical analyses were conducted using Python (version 3.9.18).

### Ethical Considerations

Data collection for the GSR-ET was centrally approved by the leading ethics committee at Ludwig-Maximilians University, Munich (689-15). Additional approvals were obtained from local ethics committees or institutional review boards at each participating center, including the ethics committee of the University of Tübingen (057/2016BO2), in accordance with local regulations. Informed consent was obtained from patients for data collection. The data used in this study were pseudonymized to ensure privacy and confidentiality. No personally identifiable information is included in the manuscript or supplementary materials. No compensation was provided to participants, as the registry relies on routine clinical data.

## Results

The results presented in this section are solely for the patients with recorded symptom onset (group A). Results for group B can be found in Multimedia Appendix 3.

### Descriptive Statistics

A total of 8467 (62.9%) patients with recorded symptom onset were included in the analysis. 4809 (56.8%) patients received intravenous thrombolysis. Table 1 shows the descriptive statistics of patients who underwent thrombolysis and patients who did not. Most of the features were statistically associated with the target variable of thrombolysis (*P*<.05).

**Table 1.** Descriptive statistics of patients with recorded symptom onset in relation to the outcome of interest.

Features	Intravenous thrombolysis (n=4809, 56.8%)	No intravenous thrombolysis (n=3658, 43.2%)	*P* value
Categorical features			
Sex, n (%)			<.001
Male	2444 (50.8)	1835 (50.2)	
Female	2364 (49.2)	1822 (49.8)	
Baseline antithrombotic medication, n (%)			
None	3042 (63.2)	2015 (55.1)	<.001
ASS^a^	1486 (31.0)	821 (22.4)	<.001
Clopidogrel	121 (2.5)	157 (4.3)	<.001
Heparins	36 (0.7)	169 (4.6)	<.001
Apixaban	25 (0.5)	165 (4.5)	<.001
Edoxaban	5 (0.1)	65 (1.8)	<.001
Rivaroxaban	21 (0.4)	134 (3.6)	<.001
Dabigatran	13 (0.3)	22 (0.6)	.002
Phenprocoumon	146 (3.0)	333 (9.1)	<.001
Others	0 (0.0)	0 (0.0)	>.99
Living status, n (%)			.002
Home	4248 (88.3)	3142 (85.9)	
Nursing home	273 (5.7)	217 (5.9)	
Nursing at home	155 (3.2)	171 (4.7)	
Comorbidity, n (%)			
Diabetes mellitus	933 (19.4)	914 (24.9)	<.001
Dyslipidemia	1868 (38.8)	1559 (42.6)	.001
Arterial hypertension	3486 (72.5)	2872 (78.5)	<.001
Atrial fibrillation	1494 (31.1)	1938 (52.9)	<.001
Prior stroke	350 (7.3)	488 (13.3)	<.001
Smoking, n (%)			.004
Nonsmoker	3200 (66.5)	2447 (66.9)	
Current smoker	808 (16.8)	533 (14.8)	
Previous smoker	447 (9.3)	395 (10.7)	
Imaging, n (%)			
Noncontrast enhanced computed tomography	4529 (94.2)	3266 (89.3)	<.001
CTA^b^	4265 (88.7)	3079 (84.2)	.66
CT^c^ perfusion	2366 (49.2)	1780 (48.7)	.03
Magnetic resonance imaging (MRI)	225 (4.7)	231 (6.3)	<.001
Magnetic resonance angiography (MRA)	170 (3.5)	166 (4.5)	.83
Magnetic resonance perfusion (MR perfusion)	87 (1.8)	90 (2.5)	.77
Occluded vessel, n (%)			
Cerebral artery – extracranial	319 (6.6)	238 (6.5)	>.99
Cerebral artery – intracranial without carotid-T involvement	280 (5.8)	212 (5.8)	.90
Cerebral artery – intracranial with carotid-T involvement	675 (14.0)	528 (14.4)	.39
Middle cerebral artery M1-segment, proximal	1514 (31.5)	1064 (29.1)	.08
Middle cerebral artery M1-segment, distal	750 (15.6)	447 (12.2)	<.001
Middle cerebral artery M2-segment	858 (17.8)	602 (16.4)	.22
Anterior cerebral artery (ACA)	146 (3.0)	93 (2.5)	.25
Posterior cerebral artery (PCA)	164 (3.4)	110 (3.0)	.41
Basilar artery (BA)	357 (7.4)	342 (9.3)	.001
Vascular artery (VA)	73 (1.5)	64 (1.7)	.39
Occluded vessel side, n (%)			<.001
left	2203 (45.8)	1611 (44.0)	
right	2126 (44.2)	1536 (41.9)	
bilateral	40 (0.8)	54 (1.5)	
Not applicable (eg basilar artery)	276 (5.7)	290 (7.9)	
Tandem stenosis, n (%)	544 (11.3)	335 (9.1)	.45
Ordinal features			
Imaging aspects, n (%)			<.001
10	1810 (37.6)	1063 (29.0)	
Not applicable (eg, basilar artery)	542 (11.2)	614 (16.8)	
9	784 (16.3)	559 (15.2)	
8	640 (13.3)	477 (13.0)	
7	394 (8.2)	324 (8.8)	
6	211 (4.4)	176 (4.8)	
5	132 (2.7)	123 (3.4)	
4	53 (1.1)	51 (1.4)	
3	20 (0.4)	32 (0.9)	
2	11 (0.2)	11 (0.3)	
1	12 (0.2)	4 (0.1)	
Thrombolysis in Cerebral Infarction score (TICI) on CTA or MRA, n (%)			<.001
0	4100 (85.2)	2998 (81.9)	
1	220 (4.6)	147 (4.0)	
2a	80 (1.6)	69 (1.9)	
2b	38 (0.8)	36 (0.9)	
3	43 (0.9)	30 (0.8)	
Not applicable	189 (3.9)	219 (5.9)	
Pre-stroke modified Rankin Scale score, median (IQR)	0 (0‐1)	0 (0‐2)	<.001
National Institute of Health Stroke Scale on admission, median (IQR)	14 (8‐18)	13 (7‐18)	.007
Continuous features			
Age (years), median (IQR*)*	75 (63‐82)	77 (66‐83)	.001
Systolic blood pressure, median (IQR)	150 (135‐170)	150 (132‐170)	.13
Diastolic blood pressure, median (IQR)	80 (72‐92)	80 (70‐92)	.32
Time between symptom onset and admission, median (IQR)	93 (56‐182)	135 (65‐255.75)	<.001

a ASS: acetylsalicylic acid.

b CTA: computed tomography angiography.

c CT: computed tomography.

### Machine Learning Model

Table 2 summarizes the performance of different ML classification models on our dataset and shows similar performance of the first 3 models across the 3 features sets (see Multimedia Appendix 4 for complete table). Despite comparable performance with LGBMClassifier, we selected the random forest model for subsequent data analysis due to its built-in feature importance metrics, which facilitate clinical interpretation, and its robustness to overfitting in datasets with noise or imbalance. In addition, since our study focuses on identifying key clinical features, random forest’s ability to effectively handle complex feature interactions makes it well-suited for our goals [44].

**Table 2.** Performance metrics of various machine learning classification models.

Model	AUROC^a^	Accuracy	*F*_1_-score
RandomForestClassifier	0.71	0.72	0.72
LGBMClassifier	0.71	0.72	0.72
AdaBoostClassifier	0.70	0.72	0.71
LogisticRegression	0.70	0.72	0.71

a AUROC: area under the receiver operating characteristic curve

### Model Performance Assessment and Interpretability

Our model was evaluated for its performance on the different feature sets and subpopulations and predicted the treatment outcome. Table 3 shows the performance metrics for patients with recorded symptom onset.

**Figure 2.**
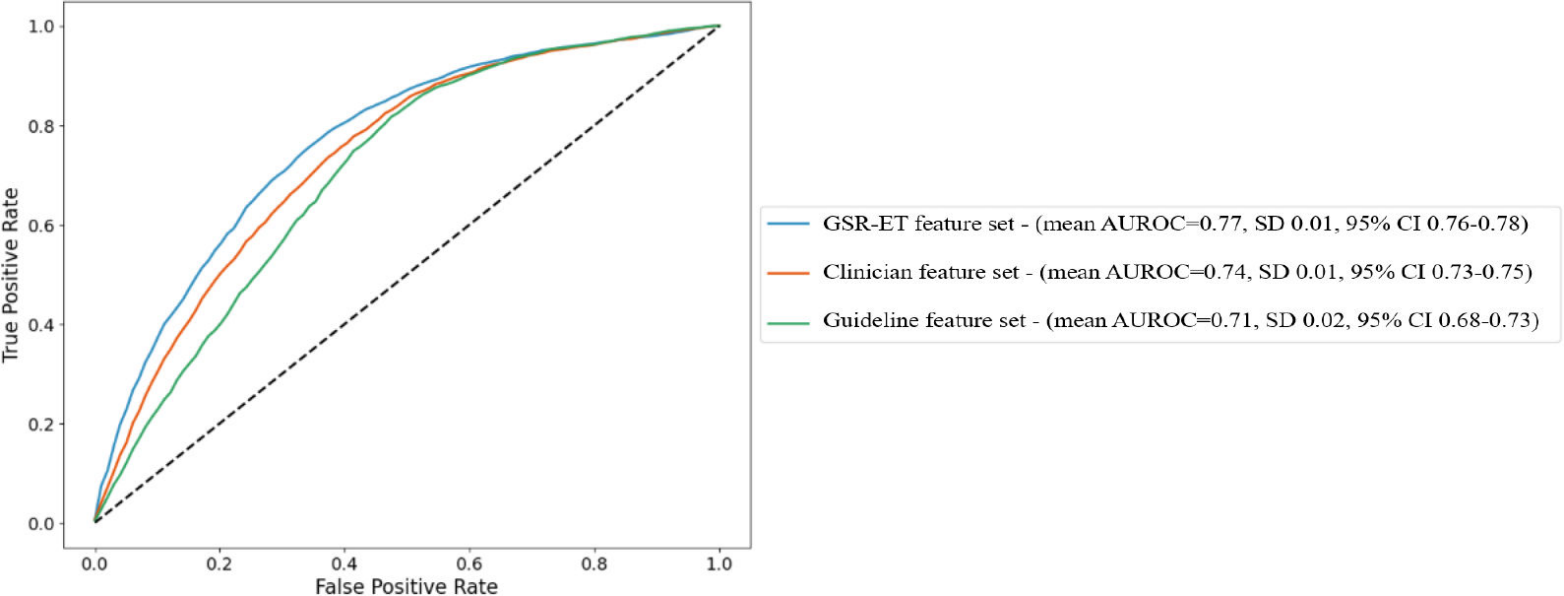
Mean area under the receiver operating characteristic curve (AUROC) across all 3 feature sets for patients with recorded symptom onset. GSR-ET: German Stroke Registry – Endovascular Treatment.

Figure 2 shows the AUROC for all feature sets: guideline, clinician and GSR-ET, with each curve representing the mean AUROC across 5 cross-validation folds in the given feature set. The GSR-ET feature set achieved the highest mean AUROC of 0.77 (SD 0.01), followed by the clinician feature set with 0.74 (SD 0.01), and the guideline feature set with 0.71 (SD 0.01). Multimedia Appendix 5 shows the plot consisting of the individual k-fold results. There was no significant improvement in performance after applying Synthetic Minority Oversampling Technique (SMOTE), indicating that class imbalance had minimal impact on the model performance. The imputation methods yielded similar results (eg, for the GSR-ET feature set, mean imputation AUROC: 0.75, k-nearest neighbor AUROC: 0.75, and multivariate imputation by chained equations: 0.77), thereby improving over the approach of dropping rows with missing values (AUROC: 0.66).

**Figure 3.**
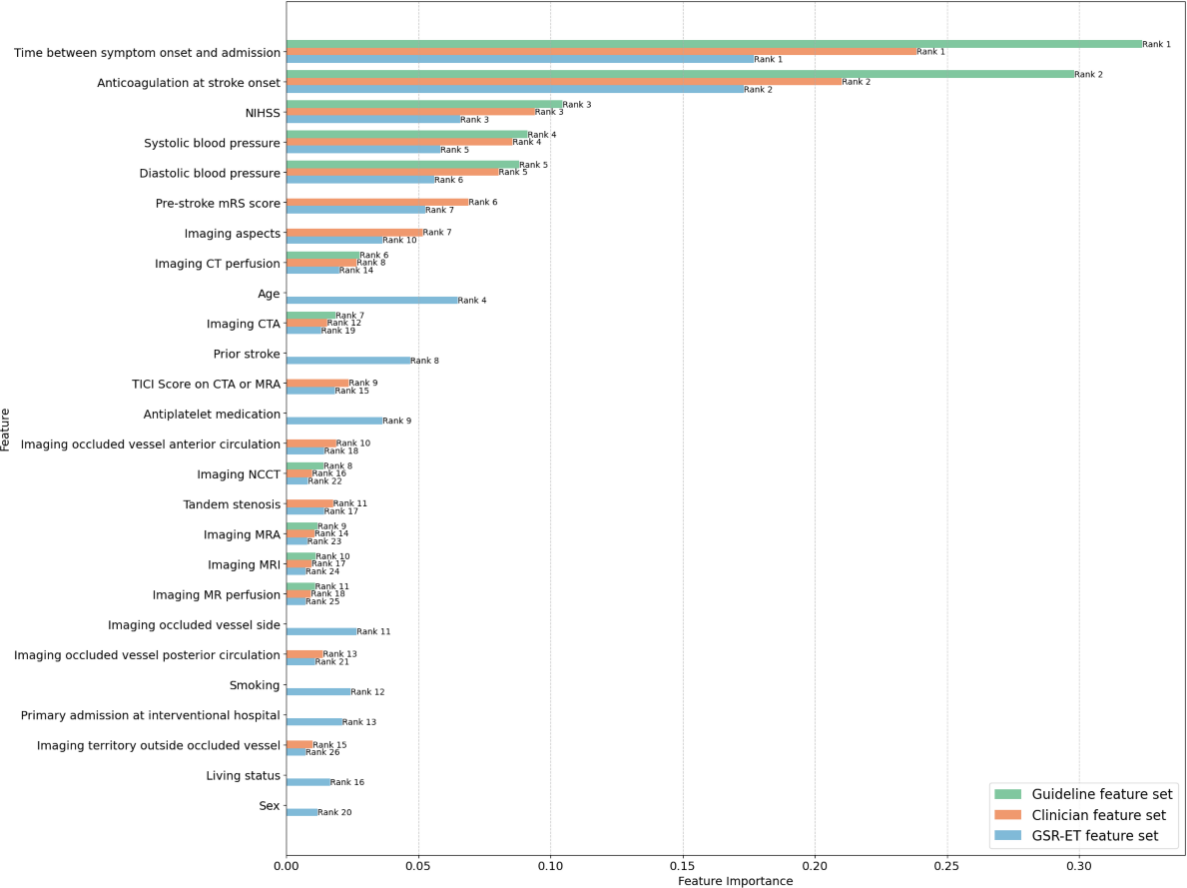
Feature importances for each feature set for patients with recorded symptom onset. CT: computed tomography; CTA: computed tomography angiography; GSR-ET: German Stroke Registry – Endovascular Treatment; MR: magnetic resonance; MRA: magnetic resonance angiography; NCCT: noncontrast computed tomography; NIHSS: National Institute of Health Stroke Scale; TICI: Thrombolysis in Cerebral Infarction.

Figures3  4 show the feature importance and permutation feature importance for each feature set, with their corresponding importance scores and rankings. The annotations on the bars represent the feature’s rank within its respective feature set. These plots highlight the varying significance of features depending on the feature set used during prediction (See Multimedia Appendix 5 for feature importance plot with confidence intervals).

The top 10 features in all 3 feature sets in both plots were mostly similar, however with differences in their rankings within the respective feature set. The “Time between symptom onset and admission” and “Anticoagulation at stroke onset” emerged as the most important features across all sets (ranked number 1 and 2 respectively). Other significant features included “NIHSS,” “Age,” “Imaging aspects,” “Pre-stroke mRS score” and “blood pressure,” all of which had high importance scores in both plots. Less influential features included various imaging modalities, “Living status” and “Sex,*”* with consistently low importance rankings across feature sets in the feature importance plot.

As seen in the beeswarm plots (Figures 5-7), the SHAP analysis further confirmed the findings provided by the random forest feature importance and permutation feature importance analyses. Each point represents a specific observation’s SHAP value, with colors corresponding to feature values (blue [cooler] color=lower value, red [warmer] color=higher value). The features are ranked in terms of mean absolute SHAP values indicating importance from the top (most important) to the bottom (least important).

The plots show each feature’s impact on the model’s prediction of the patients who did not receive intravenous thrombolysis. *“*Anticoagulation at stroke onset*”* and *“*Time between symptom onset and admission*”* have the highest SHAP values, indicating they strongly influence predictions for patients not receiving intravenous thrombolysis. Specifically, higher values of *“*Time between symptom onset and admission*”* (shown in red) push the model towards predicting no intravenous thrombolysis. Similarly, *“*Pre-stroke mRS score*”* and *“*NIHHS*”* and *“*Prior stroke” (in the GSR-ET set) also have high SHAP values. Higher scores for these features increase the likelihood of predicting no intravenous thrombolysis administration.

**Table 3.** Performance metrics of the random forest model on all 3 feature sets in the subgroup of patients with known symptom onset.

Feature set	AUROC^a^, mean (SD)	Accuracy	Precision	Recall	*F*_1_-score
Guideline	0.71 (0.02)	0.70	0.69	0.85	0.76
Clinician	0.74 (0.01)	0.70	0.70	0.86	0.77
GSR-ET	0.77 (0.01)	0.71	0.71	0.84	0.77

a AUROC: area under the receiver operating characteristic curve

**Figure 4.**
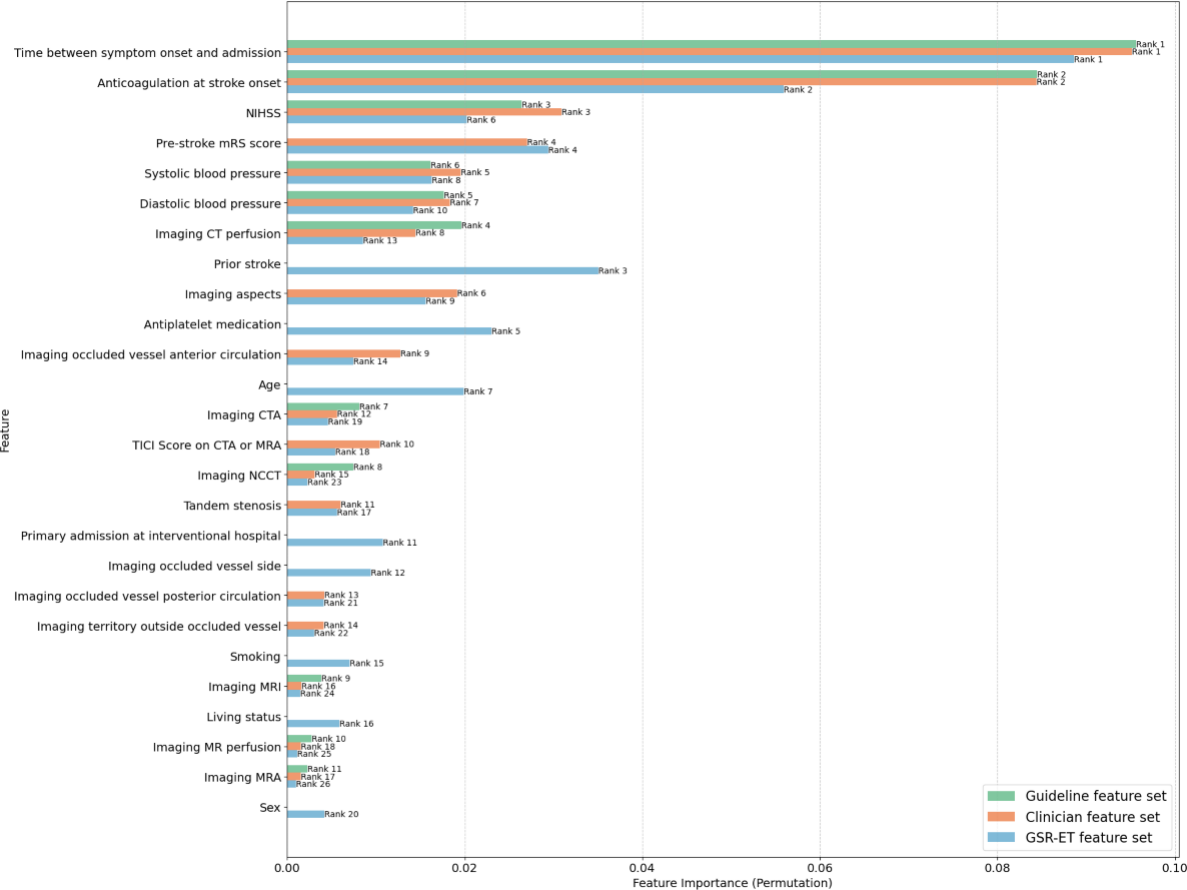
Permutation feature importances for each feature set for patients with recorded symptom onset. CT: computed tomography; CTA: computed tomography angiography; GSR-ET: German Stroke Registry – Endovascular Treatment; MR: magnetic resonance; MRA: Magnetic resonance angiography; NCCT: noncontrast computed tomography; NIHSS: National Institute of Health Stroke Scale; TICI: Thrombolysis in Cerebral Infarction;

**Figure 5.**
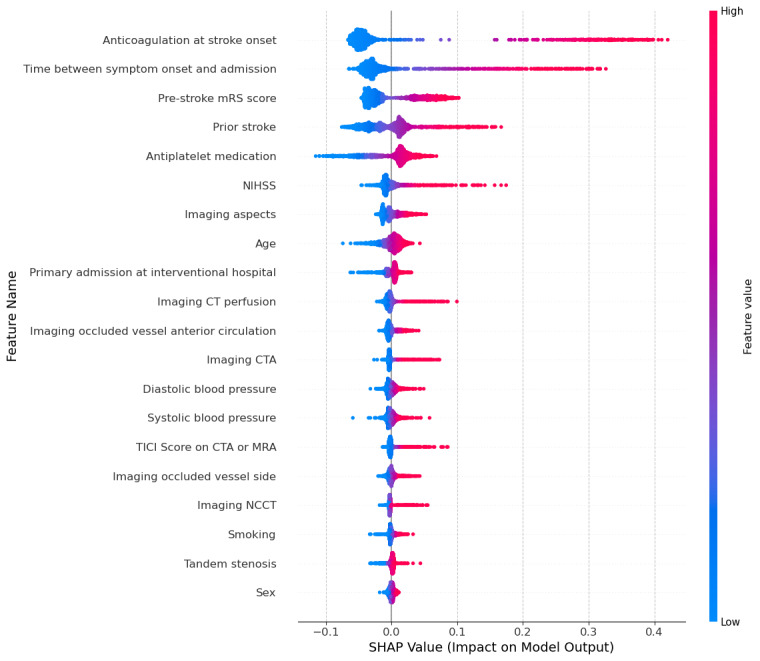
Shapley additive explanations summary plot for the German Stroke Registry – Endovascular Treatment feature set. This plot illustrates the contribution of each feature to the model’s prediction of no intravenous thrombolysis administration. Positive SHAP values increase the likelihood of predicting no intravenous thrombolysis, while negative values decrease it. Features are ranked by importance from top (most influential) to bottom (least influential). CT: computed tomography; CTA: computed tomography angiography; MRA: magnetic resonance angiography; mRS: modified Rankin Scale; NCCT: noncontrast computed tomography; NIHSS: National Institute of Health Stroke Scale; SHAP: Shapley Additive Explanations; TICI: Thrombolysis in Cerebral Infarction.

**Figure 6.**
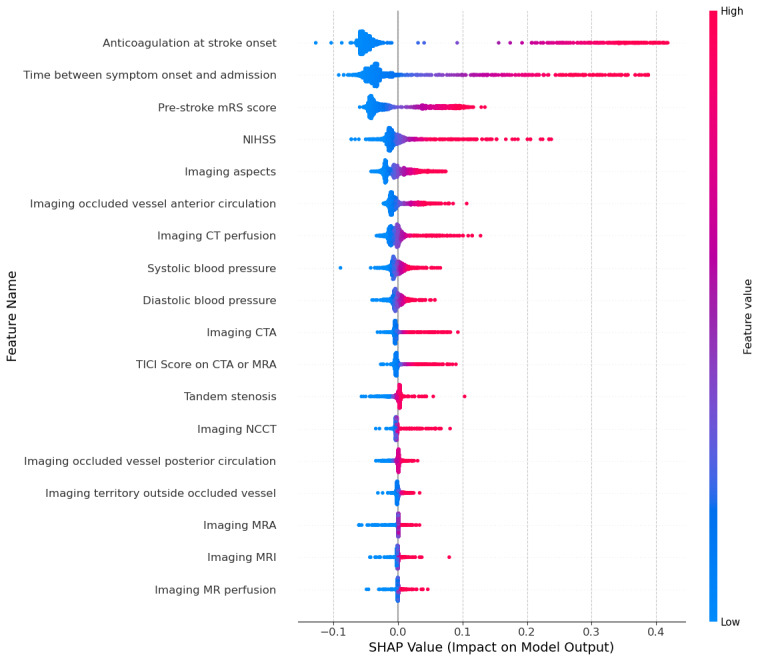
SHAP summary plot for the clinician feature set. This plot illustrates the contribution of each feature to the model’s prediction of no intravitreal administration. Positive SHAP values increase the likelihood of predicting no IVT, while negative values decrease it. Features are ranked by importance from top (most influential) to bottom (least influential). CT: computed tomography; CTA: computed tomography angiography; MR: magnetic resonance; MRA: magnetic resonance angiography; MRI: magnetic resonance imaging; mRS: modified Rankin Scale; NCCT: noncontrast computed tomography; NIHSS: National Institute of Health Stroke Scale; SHAP: Shapley Additive Explanations; TICI: Thrombolysis in Cerebral Infarction.

**Figure 7.**
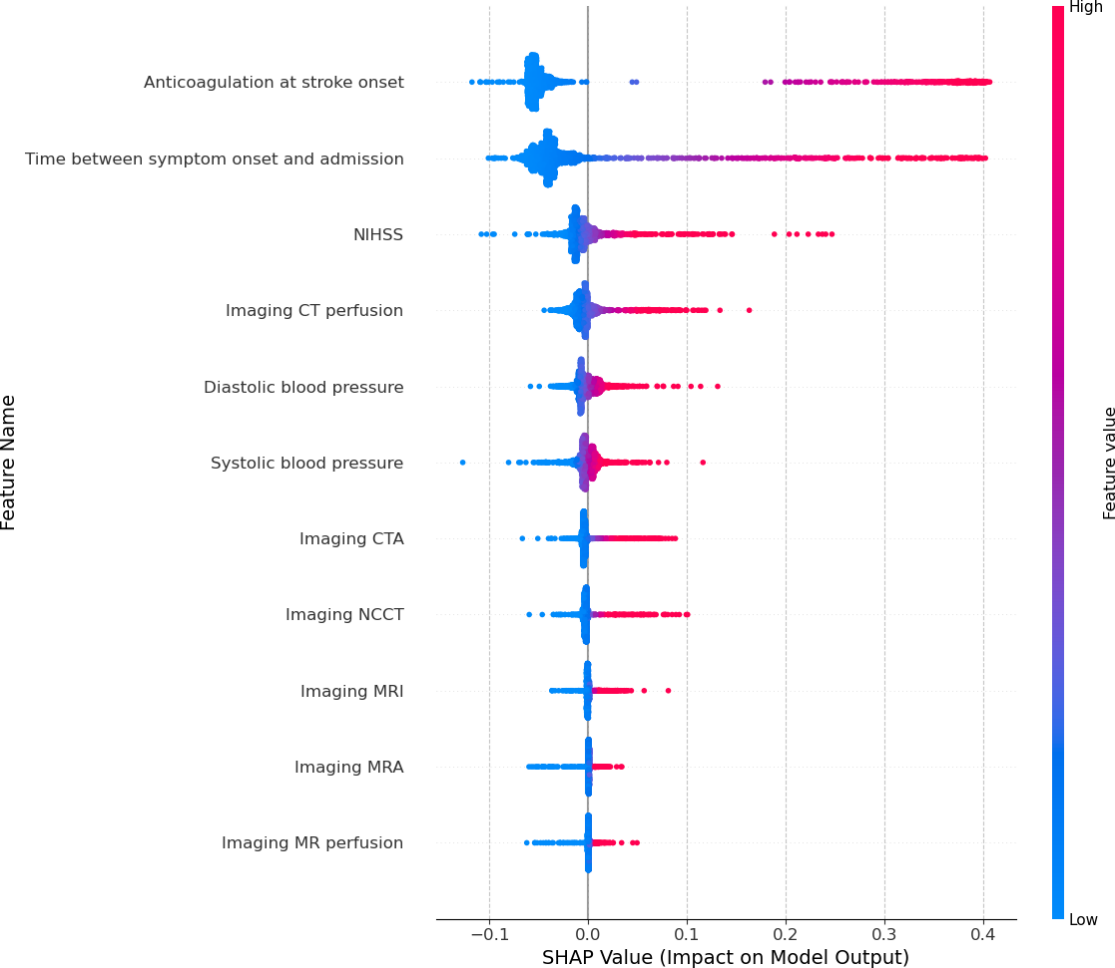
SHAP summary plot for the guideline feature set. This plot illustrates the contribution of each feature to the model’s prediction of no intravitreal administration. Positive SHAP values increase the likelihood of predicting no intravitreal, while negative values decrease it. Features are ranked by importance from top (most influential) to bottom (least influential). CT: computed tomography; CTA: computed tomography angiography; MR: magnetic resonance; MRA: magnetic resonance angiography; MRI: magnetic resonance imaging; NIHSS: National Institute of Health Stroke Scale; SHAp: Shapley Additive Explanations.

## Discussion

### Overview

The results of this study provide valuable insights into how ML models can be used to highlight discrepancies between guideline-recommended practices and clinical practice. By integrating ML with real-world registry data, we sought to identify gaps in CPGs. Specifically, we developed and tested a random forest model on three feature sets: (1) guideline set (features explicitly recommended by the German acute stroke guideline), (2) clinician set (features selected by stroke experts), and (3) the GSR-ET set, which consists of all relevant features provided in the GSR-ET that were available to treating physicians before taking the decision to initiate thrombolysis in ischemic stroke patients with large vessel occlusion undergoing endovascular reperfusion therapy.

### Principal Findings

#### Model Performance (AUROC Curves)

The AUROC curve for the 3 feature sets reveals a clear hierarchy in predictive performance. The GSR-ET feature set, which includes all reasonable features documented before thrombolysis initiation, achieved the mean AUROC curve score of 0.77. These results suggest that real-world decisions as documented in the GSR-ET incorporate additional clinical features beyond those recommended by the guidelines or even those included by stroke experts (clinician feature set), which enhances the model’s ability to predict intravenous thrombolysis administration. The relatively lower performance of the guideline feature set (AUROC=0.71) indicates that relying solely on guideline-indicated features may not be sufficient to capture the complexity of intravenous thrombolysis decision-making. This finding highlights a potential gap in the current guidelines, which may not fully account for all relevant clinical variables influencing intravenous thrombolysis administration.

#### Key Features and Possible Gaps in the German Acute Ischemic Stroke Guidelines

The top 10 features across all 3 sets were generally consistent, with the *“*Time between symptom onset and admission” emerging as the most important feature across all sets. This result underscores the critical role of timely intervention in stroke management, as shorter times from symptom onset to admission are associated with better outcomes and are a key determinant of intravenous thrombolysis eligibility [26]. “Time between symptom onset and admission” not only ranked first in all 3 feature sets but also had the greatest impact on model performance when permuted, further emphasizing its importance in predicting intravenous thrombolysis administration.

The “Pre-stroke mRS score” measures a patient’s pre-stroke functional status where a high score implies a greater level of disability, which might reduce the likelihood of receiving intravenous thrombolysis if the expected benefit is limited due to pre-existing impairment. *“*Imaging aspects*”* evaluates the extent of early ischemic changes on imaging. Lower ASPECTS scores suggest more extensive ischemic damage, potentially dissuading intravenous thrombolysis administration due to higher bleeding risk and reduced likelihood of tissue salvage despite successful reperfusion. Both features were amongst the top 10 important features in the clinician and GSR-ET feature sets. This reflects the emphasis on functional status and stroke severity in treatment decisions. These features could, together with other scores such as the NIHSS, a guideline-recommended score which assesses neurological severity of stroke (ranked 3rd in the feature importance plot), offer a comprehensive view of both current severity and pre-existing functional limitations to guide intravenous treatment decisions.

Notably, “Age,” which was not included in the guideline feature set, in line with the German acute ischemic stroke guidelines, emerged as relatively important in the GSR-ET feature set (ranked 4th). Its permutation also resulted in a drop in model performance. The SHAP values for “Age” showed that advanced age correlates with a tendency to withhold intravenous thrombolysis. This trend may reflect clinicians’ caution in treating older patients due to increased risks, such as bleeding complications or potentially lower efficacy of the treatment in older populations. While the German guidelines may not emphasize “Age” as a factor, data-driven models recognize its relevance in intravenous thrombolysis decision-making. A correlation analysis revealed a weak positive correlation (Pearson correlation coefficient=0.28) between “Age” and “Pre-stroke mRS score”, suggesting that *“*Age” is not merely an epiphenomenon but an independent feature influencing clinical decisions. This discrepancy between guideline recommendations and clinical practice points to a potential gap in the guidelines where clearer, age-specific recommendations could support clinicians, particularly given the established association between age and stroke outcomes [31,45].

Although demographic and lifestyle features such as “Smoking*,”* “Living status*,”* “Sex,” and “Prior stroke” were included in the GSR-ET feature set, their relatively lower importance scores suggest that these features may play a more indirect role in treatment determination. They contribute to the overall risk profile of stroke patients, potentially influencing clinician perception of stroke severity or recovery potential and could therefore enhance individualized assessment for long-term outcomes and guide secondary prevention strategies [46].

Patients’ use of antiplatelet and anticoagulation medications showed an influence on treatment decisions. While anticoagulation therapy is recognized as a contraindication for intravenous thrombolysis in specific scenarios, this is not explicitly detailed in the German guidelines, which instead refer clinicians to the summary of product characteristics. Similarly, the role of antiplatelet medication is less defined, as it is generally not believed to alter the benefit-risk ratio despite slightly increased bleeding risk. Our findings suggest that clinicians consider these factors, likely weighing the risks of hemorrhagic complications. A potential update to the guidelines regarding these medications could provide clinicians with granular recommendations regarding intravenous thrombolysis treatment in patients with varying antithrombotic regimens [36].

The minimal influence of “Tandem stenosis” on intravenous thrombolysis prediction suggests that the presence of concurrent stenosis in other vessels might be a significant factor in decision-making, likely due to its implications for clot accessibility and reperfusion success. This feature’s importance signals a potential need to include vascular status assessments in intravenous thrombolysis eligibility criteria within the German acute ischemic stroke guidelines.

The feature *“*Primary admission at interventional hospital*”* positively influenced the likelihood of intravenous thrombolysis treatment, suggesting that hospital resources, including the presence of specialized stroke intervention teams, play a role in treatment decisions.

Other key features such as blood pressure and imaging features consistently achieved high and minimal importance respectively across all sets. These findings reinforce the importance of managing cardiovascular risk factors and imaging in stroke patients, especially in relation to intravenous thrombolysis eligibility.

### Comparison With Previous Work

The integration of ML into clinical decision-making, especially in real-world treatment decisions, has been explored in several health care domains, but not in the context of gap search in CPGs. This section compares our approach with previous work to highlight how we contribute to the existing literature on identifying gaps in CPGs.

Though not aimed at directly refining CPGs, Abd-Alrazaq et al [47] used ML to identify gaps in COVID-19 literature. Another similar study focused on uncovering variation in clinical decision-making using registry data and ML is presented by James et al [48]. Smyth et al [14] conducted a study, using a mixed methods approach of surveys, chart reviews, and focus groups, focusing on syncope management, aiming to identify gaps between guideline-recommended care and actual clinical practice. They emphasized that guideline adherence was suboptimal, leading to overuse of some diagnostic tests while underutilizing recommended evaluations. Similarly, Bregni et al [16] tackled rectal cancer management by systematically comparing recommendations from multiple national and international CPGs and revealed the presence of “grey areas” where guidelines either lacked sufficient evidence or were not clear, resulting in variable treatment approaches. Spranger et al [17] examined the gap between hypertension guidelines and clinical practice, finding that adherence to guidelines was often inconsistent, leading to both undertreatment and overtreatment [18]. They retrospectively analyzed medical records using chart reviews and identified factors such as clinician experience and patient characteristics as major contributors to deviations from guidelines. Ebben et al [23] developed a methodology using real-world data and clinical decision trees to continuously identify and prioritize potential areas for improvements in guidelines.

Across these approaches, there is a common theme: research gaps exist across various medical fields, often driven by real-world complexities not fully captured by CPGs. Our study extends this body of work by leveraging ML not only to identify these gaps but also to quantify the predictive power of features that are often overlooked or deemphasized in CPGs. In addition, our results suggest that updating guidelines to incorporate these potentially critical features could improve treatment consistency and outcomes, similar to some of the cited studies, which advocate for guideline refinement based on empirical evidence from clinical practice.

### Limitations

While this study provides valuable insights into the potential gaps between guideline-recommended and clinician-led decision-making, several limitations must be acknowledged.

#### Registry Data

The GSR-ET consists solely of patients with a diagnosis of acute ischemic stroke, a large vessel occlusion, and patients who underwent or to whom an endovascular treatment was attempted. It is therefore important to note that within the GSR-ET, thrombolysis is only administered in conjunction with endovascular treatment but not as standalone reperfusion therapy. This means that the effect of better prediction in the GSR-ET feature set may be related to the fact that GSR-ET patients are a subset of strokes with large vessel occlusion and may therefore not reflect thrombolysis decision-making in the overall stroke patient.

#### Feature Interaction

While feature importances can identify variables that enhance the decision-making accuracy of our ML model, they do not reveal on a deeper level how these features interact with other variables in the GSR-ET. Consequently, simply knowing that a feature is influential does not clarify its optimal placement or threshold within the CPG decision framework.

#### Guideline Flexibility

CPGs are inherently flexible and are designed to provide general recommendations that accommodate a broad range of clinical scenarios. Consequently, they may not include all the possible factors that inform real-world decision-making by clinicians. Even though our study identified features not explicitly mentioned in the guidelines, it is possible that these features may still be deliberately excluded, as guideline developers may prioritize flexibility over incorporating factors that, while influential in decision-making, are not deemed essential for standardization in future guideline updates.

#### Limited Focus on Clinical Outcomes

This study focused primarily on predicting intravenous thrombolysis administration and identifying which variables were involved in decision-making but did not investigate the long-term clinical outcomes associated with these decisions. While deviations from the guidelines and independent clinical judgments were identified, it is unclear whether these decisions led to better or worse patient outcomes. This makes it difficult to assess whether the identified features should indeed be included in future guidelines based solely on the results of this study.

### Future Directions

The methodology applied in this study offers a systematic approach to uncover discrepancies between CPGs and real-world decision-making. Future research using larger datasets could strengthen these findings and support guideline refinements. Integrating such approaches into clinical workflows may enable rapid identification of guideline gaps or nonadherence, ultimately contributing to improved patient care.

### Conclusion

This study demonstrates the potential of combining ML with real-world data to systematically identify gaps between clinical practice and current CPGs. In the case of intravenous thrombolysis for large vessel occlusion stroke, factors such as age and pre-stroke disability, which are not explicitly addressed in current German guidelines, emerged as relevant to treatment decisions. These features may merit consideration in future iterations of CPGs to improve alignment with clinical realities.

Providing clear guidance on them would not only support clinicians in navigating complex patient profiles and making ethical, patient-centered decisions but also enhance their moral confidence and potentially offer legal protection. Specifically, future CPGs may emphasize that advanced age alone should exclude patients from thrombolysis, as it can enable recovery to pre-stroke conditions. However, a severely impaired pre-stroke functional status, particularly in advanced age, may not always represent a meaningful therapeutic goal.

## Supplementary material

10.2196/69282 Multimedia Appendix 1 Detailed list of the criteria used to exclude patients from the analysis.

10.2196/69282 Multimedia Appendix 2 Feature sets used in comparing predictive performance.

10.2196/69282 Multimedia Appendix 3 Results of group B – patient subgroup with recorded “last seen well” or “time of symptom recognition”

10.2196/69282 Multimedia Appendix 4 Complete classification model comparison table.

10.2196/69282 Multimedia Appendix 5 Additional results for group A – patients with recorded symptom onset.

## Abbreviations

ASPECTS: Alberta Stroke Program Early CT Score
AUROC: area under the receiver operating characteristic curve
CPG: clinical practice guideline
CT: computed tomography
CTA: computed tomography angiography
GSR-ET: German Stroke Registry – Endovascular Treatment
ML: machine learning
MRA: magnetic resonance angiography
NCCT: noncontrast computed tomography
NIHSS: National Institute of Health Stroke Scale
SHAP: Shapley Additive Explanations
SMOTE: Synthetic Minority Oversampling Technique
TICI: Thrombolysis in Cerebral Infarction
