# Supplementary material for: Combining Machine Learning With Real-World Data to Identify Gaps in Clinical Practice Guidelines: Feasibility Study Using the Prospective German Stroke Registry and the National Acute Ischemic Stroke Guidelines
Source: JMIR Med Inform. 2025 Jul 11;13:e69282. doi: 10.2196/69282 (PMC12274016; doi:10.2196/69282)
Supplement: Multimedia Appendix 1 [file medinform-v13-e69282-s001.pdf]

Supplementary Material 1

This list consists of the criteria used to exclude patients including entries deemed implausible (Implausible information as described in the flow chart)

1. Patients less than 18 years old.
2. Patients with no information on intravenous thrombolysis administration.
3. Patients missing time variables (no symptom onset, no "last seen well" time, and no "time of recognition").
4. Patients where the "last seen well" time is the same as "time of recognition".
5. Patients who, according to the data, did not receive intravenous thrombolysis but received intravenous thrombolysis treatment on-site.
6. Patients treated at an interventional hospital, where the difference between admission time and intravenous thrombolysis administration time is less than 0.
7. Patients not treated at an interventional hospital, where the difference between admission time and intravenous thrombolysis administration time is less than 0, and no imaging was performed at the interventional hospital.
8. Patients who did not receive intravenous thrombolysis but underwent a "drip and ship" process.
9. Patients who received intravenous thrombolysis on site, but the difference between admission time and intravenous thrombolysis administration time is less than 0.
10. Patients who did not receive intravenous thrombolysis on site, but the difference between admission time and intravenous thrombolysis administration time is greater than 0.
11. Patients who received intravenous thrombolysis on site, but the difference between admission time and intravenous thrombolysis administration time is equal to 0.
12. Patients who did not receive intravenous thrombolysis on site, but the difference between admission time and intravenous thrombolysis administration time is equal to 0.
13. Patients with no information on intravenous thrombolysis treatment on site, but the difference between admission time and intravenous thrombolysis administration time is less than 0.
14. Patients with no information on intravenous thrombolysis treatment on site, but the difference between admission time and intravenous thrombolysis administration time is greater than 0.
