# Supplementary material for: Combining Machine Learning With Real-World Data to Identify Gaps in Clinical Practice Guidelines: Feasibility Study Using the Prospective German Stroke Registry and the National Acute Ischemic Stroke Guidelines
Source: JMIR Med Inform. 2025 Jul 11;13:e69282. doi: 10.2196/69282 (PMC12274016; doi:10.2196/69282)
Supplement: Multimedia Appendix 2 [file medinform-v13-e69282-s002.pdf]

Supplementary Material 2

Feature sets used in comparing predictive performance using random forest

Feature	Description	German Stroke Registry features	Clinician features	Guideline Features
Age	Age on admission	✓	✗	✗
Sex	Patient's sex	✓	✓	✗
Aspirin	Baseline medication aspirin	✓	✗	✗
Clopidogrel	Baseline medication clopidogrel	✓	✗	✗
Aggrenox	Baseline medication aggrenox	✓	✗	✗
Heparins	Baseline medication heparins	✓	✓	✓
Apixaban	Baseline medication apixaban	✓	✓	✓
Rivaroxaban	Baseline medication Rivaroxaban	✓	✓	✓
Dabigatran	Baseline medication dabigatran	✓	✓	✓
Edoxaban	Baseline medication edoxaban	✓	✓	✓
Phenprocoumon	Baseline medication phenprocoumon	✓	✓	✓
Living status	Patient's living status on admission	✓	✗	✗

Pre-stroke Modified Rankin Score	Pre-stroke Modified Rankin Score on admission	✓	✓	✗
National Institutes of Health Stroke Scale Score (NIHSS)	National Institutes of Health Stroke Scale Score (NIHSS) on admission	✓	✓	✓
Blood pressure on admission systolic	Blood pressure on admission systolic [mmHg]	✓	✓	✓
Blood pressure on admission diastolic	Blood pressure on admission diastolic [mmHg]	✓	✓	✓
Comorbidity previous stroke	Comorbidity on admission – previous stroke	✓	✗	✗
Imaging ncct	Patient underwent Cranial Non-contrast Computed Tomography (NCCT)	✓	✓	✓
Imaging cta	Computer Tomography Angiography (CTA) performed on admission	✓	✓	✓
Imaging ctperf	Computed Tomography (CT) perfusion performed on admission	✓	✓	✓
Imaging mri	Patient underwent Magnetic Resonance Imaging (MRI)	✓	✓	✓
Imaging mra	Magnetic Resonance Angiography (MRA) performed on admission	✓	✓	✓

Imaging mrperf	Magnetic Resonance (MR) perfusion performed on admission	✓	✓	✓
Imaging aspects	count of Alberta Stroke Program Early CT score	✓	✓	✗
Imaging occluded vessel BA	occluded vessel based on the results of the cerebral imaging on admission - Basilar artery	✓	✓	✗
Imaging occluded vessel VA	occluded vessel based on the results of the cerebral imaging on admission - Vascular artery	✓	✓	✗
Imaging occluded vessel PCA	occluded vessel based on the results of the cerebral imaging on admission - Posterior cerebral artery	✓	✓	✗
Imaging occluded vessel ACA	occluded vessel based on the results of the cerebral imaging on admission - Anterior cerebral artery	✓	✓	✗
Imaging occluded vessel CAextra	occluded vessel based on the results of the cerebral imaging on admission – extracranial	✓	✓	✗
Imaging occluded vessel CAintraT	occluded vessel based on the results of the cerebral imaging on admission – Cerebral artery intracranial with carotid-T involvement	✓	✓	✗
Imaging occluded vessel CAintranoT	occluded vessel based on the results of the cerebral imaging on admission –	✓	✓	✗

	Cerebral artery intracranial without carotid-T involvement			
Imaging occluded vessel MCAm1prox	occluded vessel based on the results of the cerebral imaging on admission - middle cerebral artery m1 proximal	✓	✓	✗
Imaging occluded vessel MCAm1dist	occluded vessel based on the results of the cerebral imaging on admission - middle cerebral artery m1 distal	✓	✓	✗
Imaging occluded vessel MCAm2	occluded vessel based on the results of the cerebral imaging on admission - middle cerebral artery m2	✓	✓	✗
Imaging occluded vessel side	side of vessel occlusion based on the results of the cerebral imaging	✓	✓	✗
Imaging occluded vessel tici	Thrombolysis in Cerebral Infection (TICI) grade based on the results of the cerebral imaging	✓	✓	✗
Treat extracranial stent yes	Treatment of the A. carotis stenosis, extracranial, with stenting or PTA	✓	✓	✗
Time between admission and symptom onset	Time between admission and symptom onset	✓	✓	✓
Time between last seen well and symptom onset	Time between last seen well and symptom onset	✓	✓	✓

Time between time of recognition and symptom onset	Time between time of recognition and symptom onset	✓	✓	✓
Intravenous thrombolysis	Intravenous thrombolysis performed	✓	✓	✓
