# Supplementary material for: Combining Machine Learning With Real-World Data to Identify Gaps in Clinical Practice Guidelines: Feasibility Study Using the Prospective German Stroke Registry and the National Acute Ischemic Stroke Guidelines
Source: JMIR Med Inform. 2025 Jul 11;13:e69282. doi: 10.2196/69282 (PMC12274016; doi:10.2196/69282)
Supplement: Multimedia Appendix 3 [file medinform-v13-e69282-s003.pdf]

Supplementary Material 3

Group B: Results of patient sub-group with recorded “last seen well” and/or “time of symptom recognition”

Table A: Descriptive statistics of patients with recorded “last seen well” and/or “time of symptom recognition” in relation to the outcome of interest

		**Intravenous thrombolysis (n = 1,568, 32%)**	**No intravenous thrombolysis (n = 3,405, 68%)**	***P*-value**
**Categorical features**				
Sex, n (%)	Male	742 (47%)	1,460 (43%)	.004
	Female	826 (53%)	1,944 (57%)	
Baseline antithrombotic medication, n (%)	none	978 (62%)	2,092 (62%)	.646
	ASS	487 (32%)	810 (24%)	<.001
	Clopidogrel	37 (2%)	99 (3%)	<.001
	Heparins	25 (2%)	79 (2%)	<.001
	Apixaban	3 (0.2%)	106 (3%)	<.001
	Edoxaban	1 (0.07%)	27 (1%)	<.001
	Rivaroxaban	7 (0.5%)	75 (2%)	<.001
	Dabigatran	3 (0.2%)	14 (0.4%)	.031
	Phenprocoumon	54 (3%)	225 (6%)	<.001
	Others	0 (0%)	0 (0%)	1
Living status, n (%)	Home	1,309 (85%)	2,754 (83%)	.002
	Nursing home	160 (10%)	297 (9%)	
	Nursing at home	66 (4%)	268 (8%)	
Comorbidity, n (%)	Diabetes mellitus	341 (22%)	817 (24%)	<.001
	Dyslipidemia	613 (40%)	1,482 (44%)	.001
	Arterial hypertension	1,163 (75%)	2,644 (79%)	<.001
	Atrial fibrillation	491 (32%)	1,619 (48%)	<.001
	Prior stroke	125 (15%)	380 (19%)	<.001
Smoking, n (%)	Non-smoker	1,057 (74%)	2,258 (72 %)	.004

	Current smoker	221 (15%)	502 (16%)	
	Previous smoker	155 (11%)	356 (11%)	
Imaging, n (%)	Non-contrast enhanced computed tomography (NCCT)	1,407 (93%)	2,995 (92%)	<.001
	Computed tomography angiography (CTA)	1,291 (92%)	2,843 (95%)	.659
	Computed tomography Perfusion (CT perfusion)	821 (58%)	1,895 (63%)	.026
	Magnetic resonance imaging (MRI)	148 (10%)	337 (10%)	<.001
	Magnetic resonance angiography (MRA)	112 (75%)	222 (65%)	.817
	Magnetic resonance perfusion (MR perfusion)	42 (28%)	92 (27%)	.767
Occluded vessel, n (%)	Cerebral artery - extracranial	92 (6%)	232 (7%)	1
	Cerebral artery - intracranial without carotid-T involvement	92 (6%)	222 (7%)	.900
	Cerebral artery - intracranial with carotid-T involvement	242 (16%)	476 (15%)	.234
	Middle cerebral artery M1-segment, proximal	520 (34%)	1,090 (33%)	.077

	Middle cerebral artery M1-segment, distal	227 (15%)	514 (16%)	<.001
	Middle cerebral artery M2-segment	242 (16%)	529 (16%)	.216
	Anterior cerebral artery	39 (3%)	108 (3%)	.254
	Posterior cerebral artery	40 (3%)	103 (3%)	.416
	Basilar artery	116 (8%)	220 (7%)	.001
	Vascular artery	23 (2%)	43 (1%)	.386
Occluded vessel side, n (%)	left	748 (49%)	1,642 (50%)	<.001
	right	658 (43%)	1,386 (42%)	
	bilateral	13 (1%)	37 (1%)	
	Not applicable (e.g. basilar artery)	89 (6%)	184 (6%)	
Tandem stenosis, *n (%)*		193 (78%)	339 (75%)	.454
**Ordinal features**				
Imaging aspects, n (%)	10	518 (34%)	722 (22%)	<.001
	not applicable (e.g. basilar artery)	163 (10%)	477 (14%)	
	9	250 (16%)	458 (14%)	
	8	231 (15%)	546 (17%)	
	7	161 (10%)	424 (13%)	
	6	95 (6%)	258 (8%)	
	5	43 (3%)	180 (5%)	
	4	20 (1%)	89 (2%)	
	3	13 (0.8%)	40 (1%)	
	2	7 (0.5%)	22 (0.7%)	
	1	3 (0.2%)	12 (0.3%)	
Thrombolysis in cerebral infarction score	0	1,332 (88%)	2,796 (86%)	<.001
	1	76 (5%)	132 (4%)	
	2a	17 (1%)	46 (1%)	

(TICI) on CTA or MRA, n (%)	2b	15 (1%)	15 (0.5%)	
	3	12 (0.8%)	27 (0.8%)	
	Not applicable	62 (4%)	242 (7%)	
Pre-stroke modified Rankin Scale score, *median (IQR)*		0 (0 - 1)	0 (0 - 2)	<.001
National institute of health stroke scale on admission, *median (IQR)*		14 (10 - 18)	15 (10 - 19)	.007
**Continuous features**				
Age, *median (IQR)*		76 (66 - 84)	78 (68 - 85)	.001
Systolic blood pressure, *median (IQR)*		150 (133 - 170)	150 (130 - 170)	.006
Diastolic blood pressure, *median (IQR)*		80 (70 - 92)	80 (70 - 94)	.495
Time between last seen well and admission, *median (IQR)*		255 (150 - 479)	570 (309 – 832)	<.001
Time between time of recognition and admission, *median (IQR)*		86 (54.75 - 174)	114 (63 – 210)	.919

Table D: Patients with "last seen well” time and/or “time of symptom recognition”: Performance metrics of random forest model on all three feature sets

Feature Set	Mean AUROC	Overall accuracy	Precision	Recall	F1 Score
Guideline	0.74	0.77	0.62	0.49	0.55
Clinician	0.76	0.76	0.61	0.44	0.51
GSR-ET	0.77	0.77	0.67	0.43	0.52

Figure A: Mean AUROC with K-folds for patients with recorded last seen well and/or time of recognition (Group B)

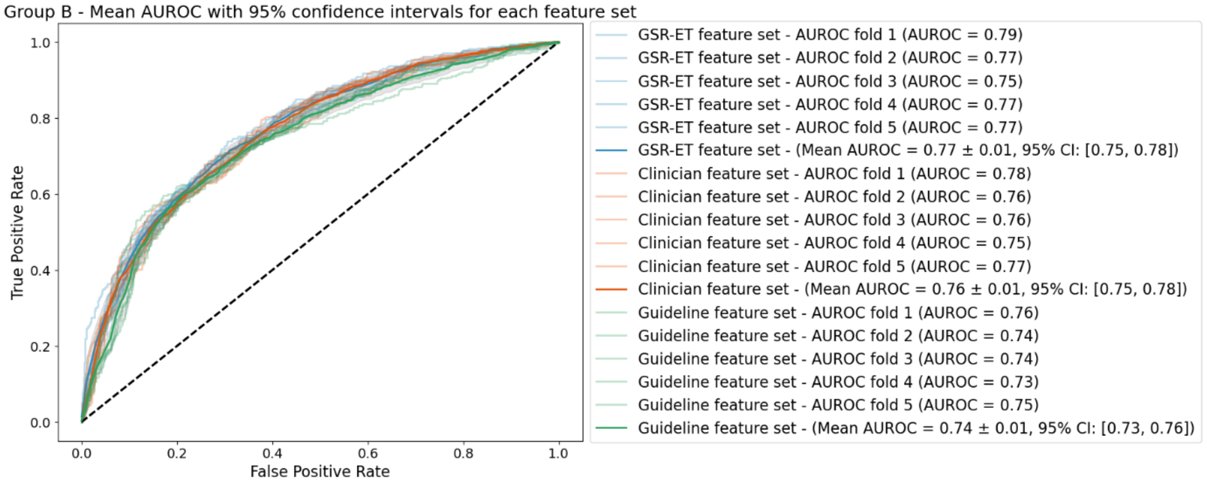

Figure B: Mean AUROC with variability for each feature set (Group B).

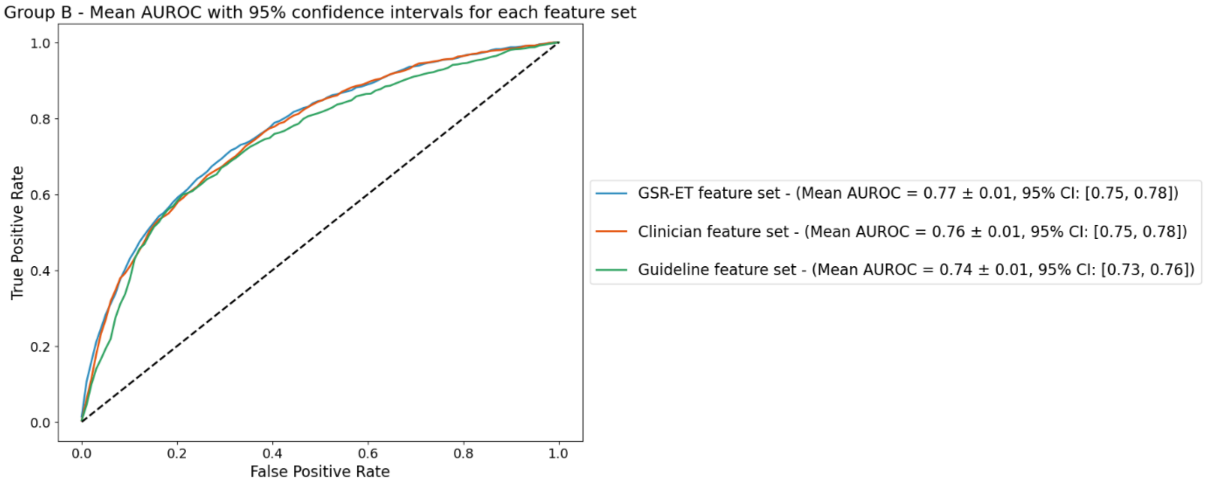

Figure C: Feature importances for each feature set for patients with recorded “last seen well” and/or “time of symptom recognition” (Group B)

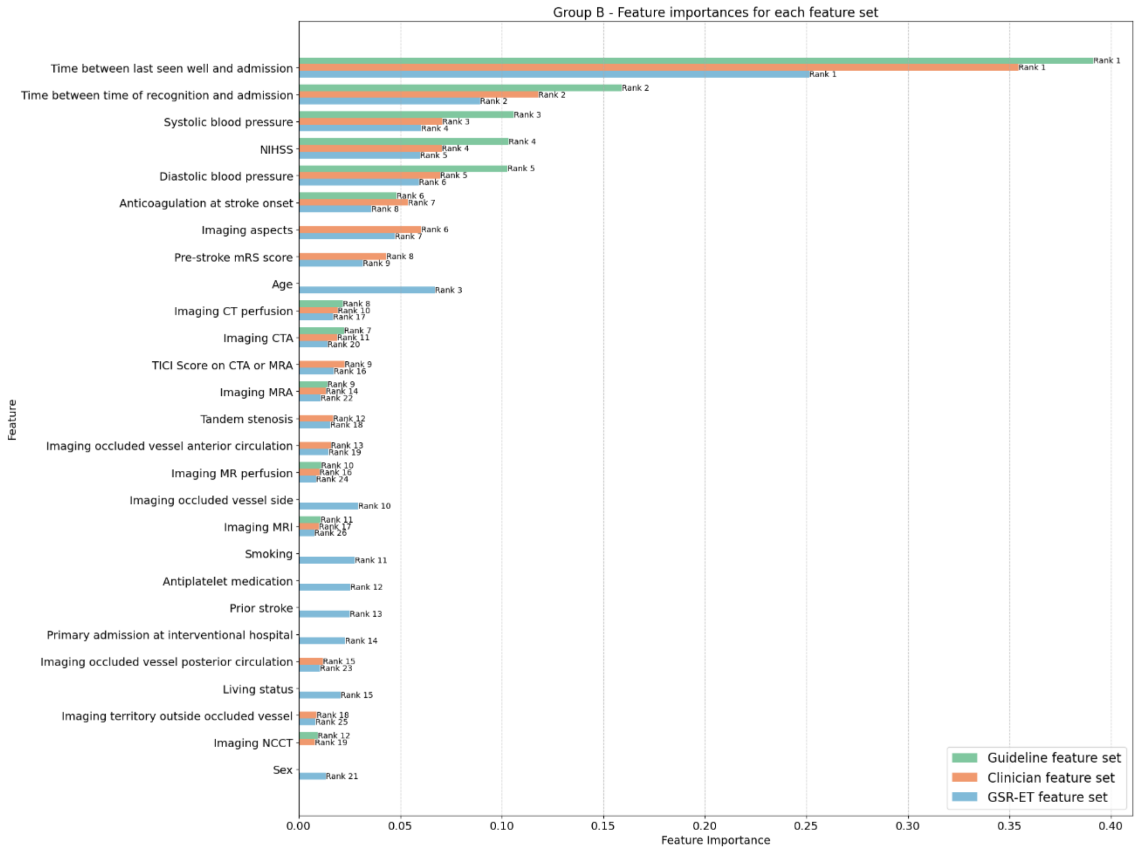

Figure D: Permutation feature importances for each feature set for patients with recorded “last seen well” and/or “time of symptom recognition” (Group B)

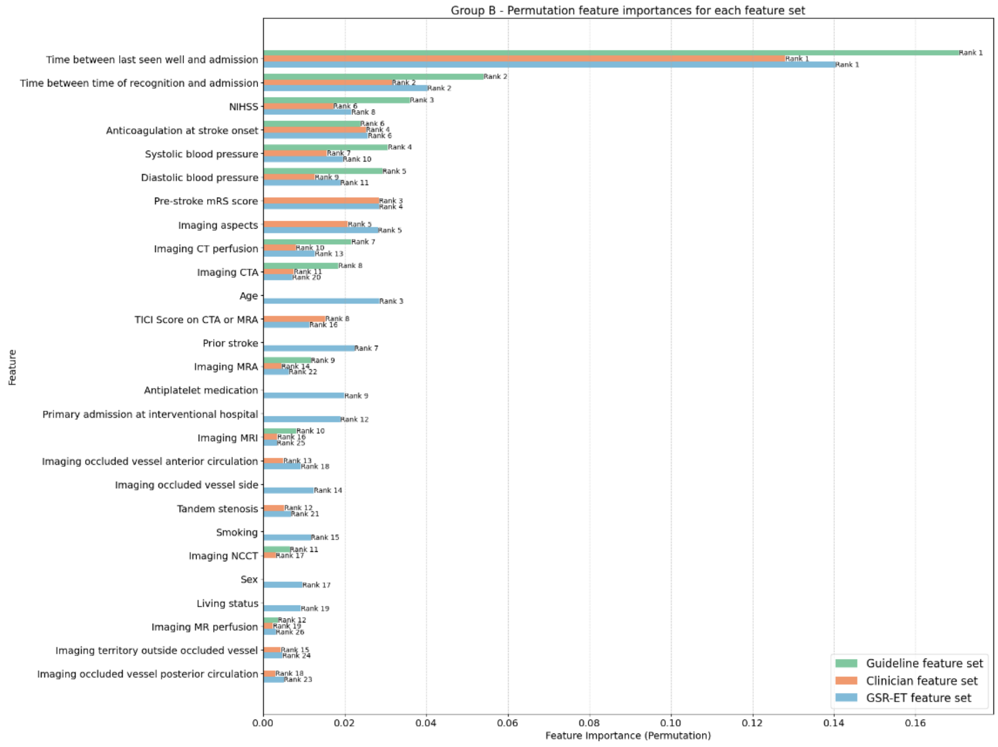

Figure F: Feature importance with confidence intervals (error bars)

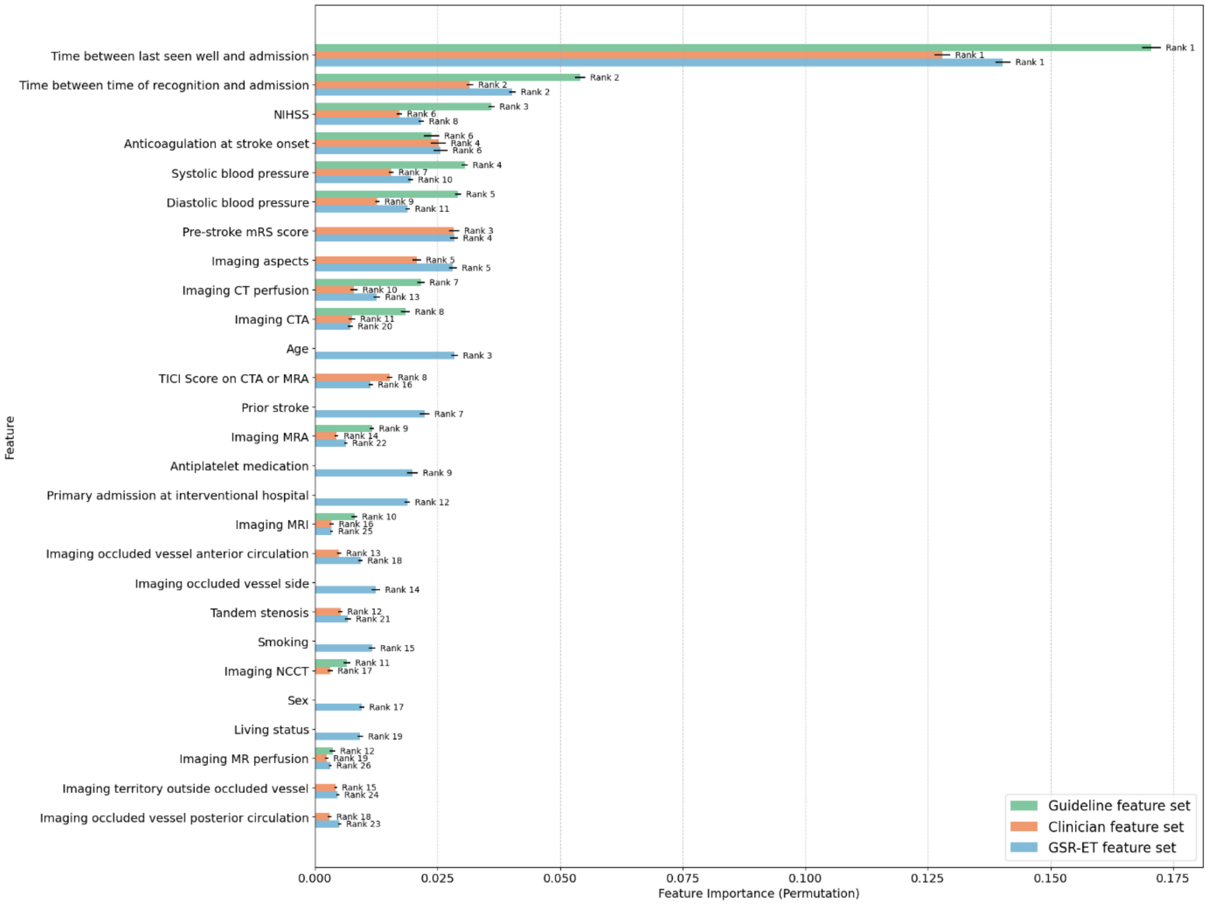

Figure F, G, H: SHAP Summary plots for all feature sets for patients with recorded “last seen well” and/or “time of symptom recognition” (Group B)

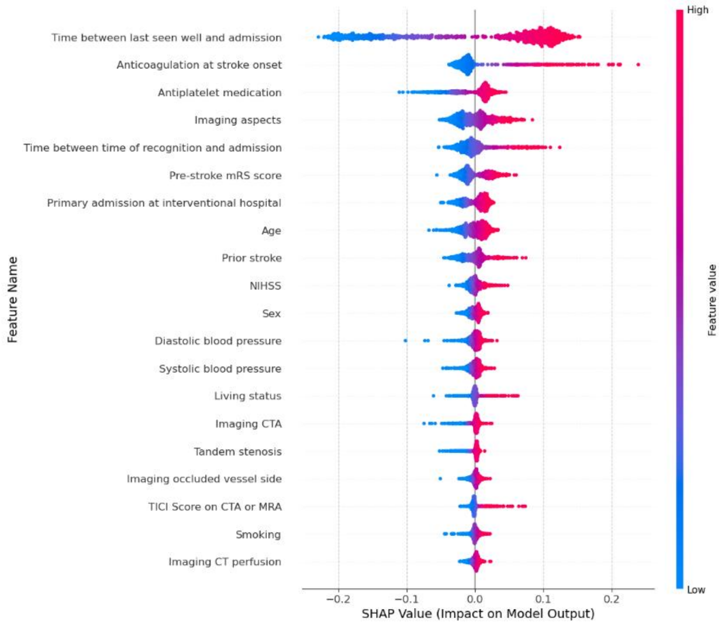

SHAP Summary Plot for Group B - GSR-ET feature set:
This plot illustrates the contribution of each feature to the model's prediction of no IVT administration.
Positive SHAP values increase the likelihood of predicting no IVT, while negative values decrease it.
Features are ranked by importance from top (most influential) to bottom (least influential).

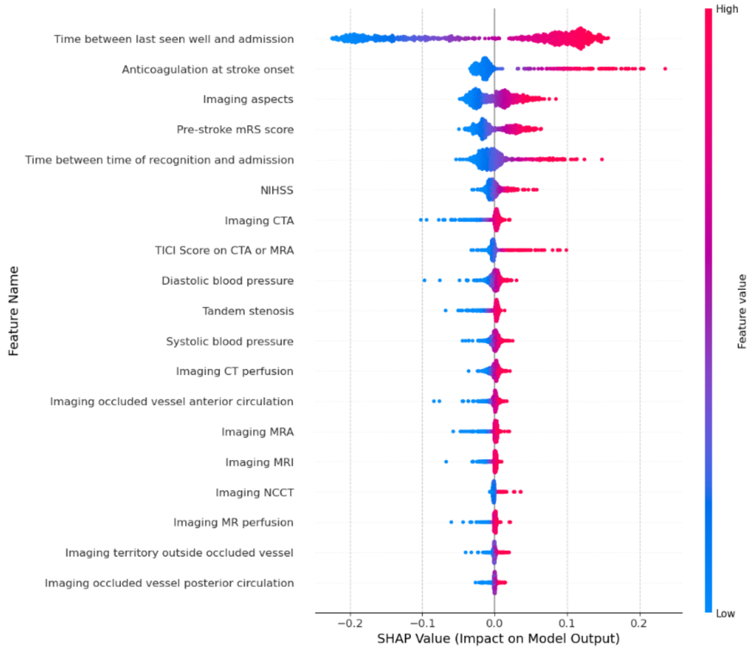

SHAP Summary Plot for Group B - Clinician feature set:
This plot illustrates the contribution of each feature to the model's prediction of no IVT administration.
Positive SHAP values increase the likelihood of predicting no IVT, while negative values decrease it.
Features are ranked by importance from top (most influential) to bottom (least influential).

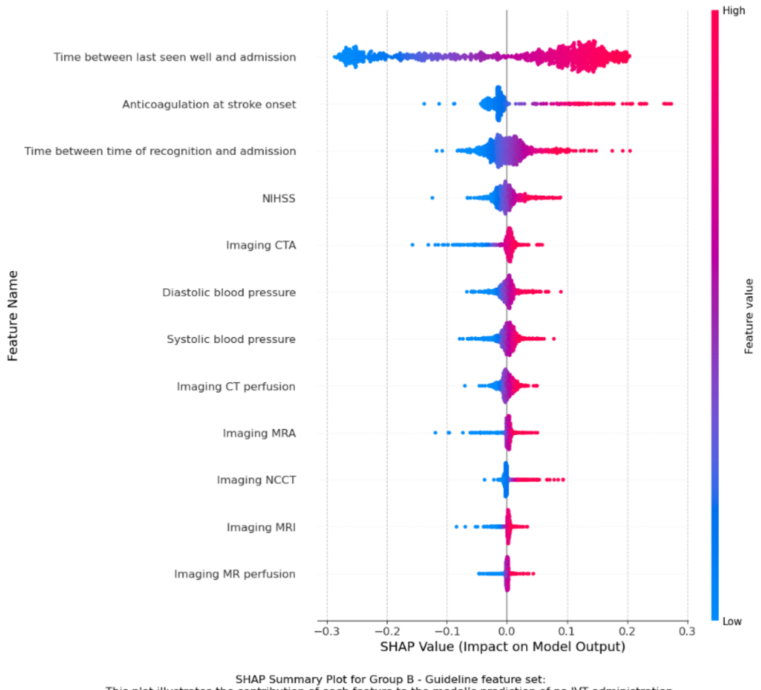

SHAP Summary Plot for Group B - Guideline feature set:
This plot illustrates the contribution of each feature to the model's prediction of no IVT administration.
Positive SHAP values increase the likelihood of predicting no IVT, while negative values decrease it.
Features are ranked by importance from top (most influential) to bottom (least influential).
