# Supplementary material for: Combining Machine Learning With Real-World Data to Identify Gaps in Clinical Practice Guidelines: Feasibility Study Using the Prospective German Stroke Registry and the National Acute Ischemic Stroke Guidelines
Source: JMIR Med Inform. 2025 Jul 11;13:e69282. doi: 10.2196/69282 (PMC12274016; doi:10.2196/69282)
Supplement: Multimedia Appendix 4 [file medinform-v13-e69282-s004.pdf]

Supplementary Material 4

Complete classification model comparison table

Model	AUC	Accuracy	F1-Score
RandomforestClassifier	0.71	0.72	0.72
LGBMClassifier	0.71	0.72	0.72
AdaBoostClassifier	0.70	0.72	0.71
LogisticRegression	0.70	0.72	0.71
CalibratedClassifierCV	0.70	0.72	0.71
XGBClassifier	0.70	0.71	0.71
LinearSVC	0.70	0.72	0.71
BaggingClassifier	0.70	0.70	0.70
LinearDiscriminantAnalysis	0.70	0.72	0.71
RidgeClassifierCV	0.70	0.72	0.71
RidgeClassifier	0.70	0.72	0.71
NuSVC	0.69	0.70	0.70
ExtraTreesClassifier	0.69	0.70	0.70
SVC	0.68	0.70	0.69
SGDClassifier	0.66	0.66	0.66
NearestCentroid	0.65	0.66	0.66
DecisionTreeClassifier	0.65	0.66	0.66
BernoulliNB	0.64	0.65	0.65
PassiveAggressiveClassifier	0.64	0.65	0.64
Perceptron	0.61	0.63	0.62
GaussianNB	0.61	0.64	0.61
KNeighborsClassifier	0.59	0.61	0.60
ExtraTreeClassifier	0.59	0.60	0.60
QuadraticDiscriminantAnalysis	0.56	0.54	0.52
LabelSpreading	0.53	0.48	0.40
LabelPropagation	0.53	0.48	0.40
DummyClassifier	0.50	0.56	0.41
