# Supplementary material for: Combining Machine Learning With Real-World Data to Identify Gaps in Clinical Practice Guidelines: Feasibility Study Using the Prospective German Stroke Registry and the National Acute Ischemic Stroke Guidelines
Source: JMIR Med Inform. 2025 Jul 11;13:e69282. doi: 10.2196/69282 (PMC12274016; doi:10.2196/69282)

Supplementary Material 5

Group A: Patient sub-group with recorded symptom onset results.

Figure A: Mean AUROC with k-fold

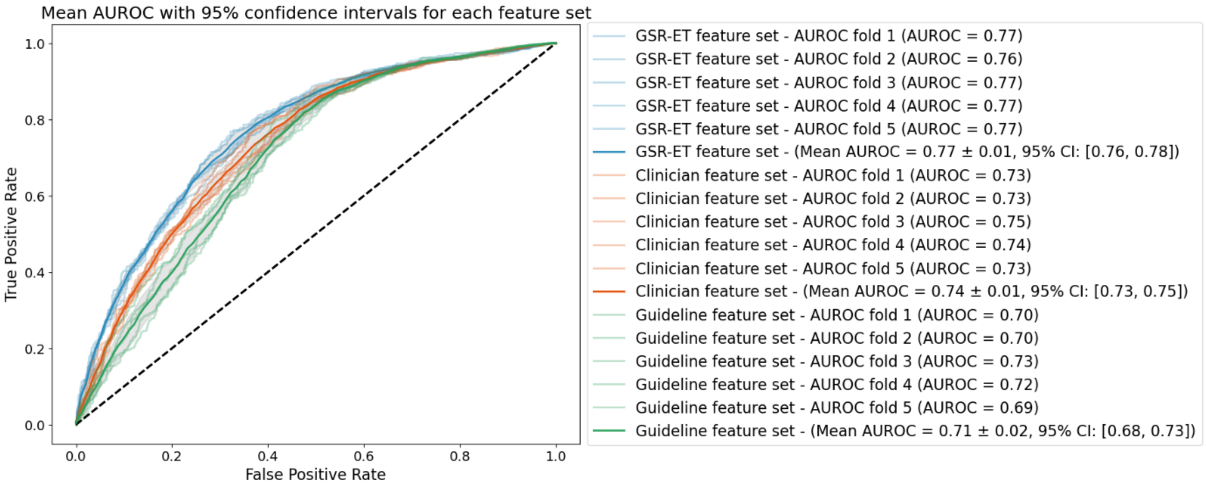

Figure B: Feature importance with confidence intervals (error bars).

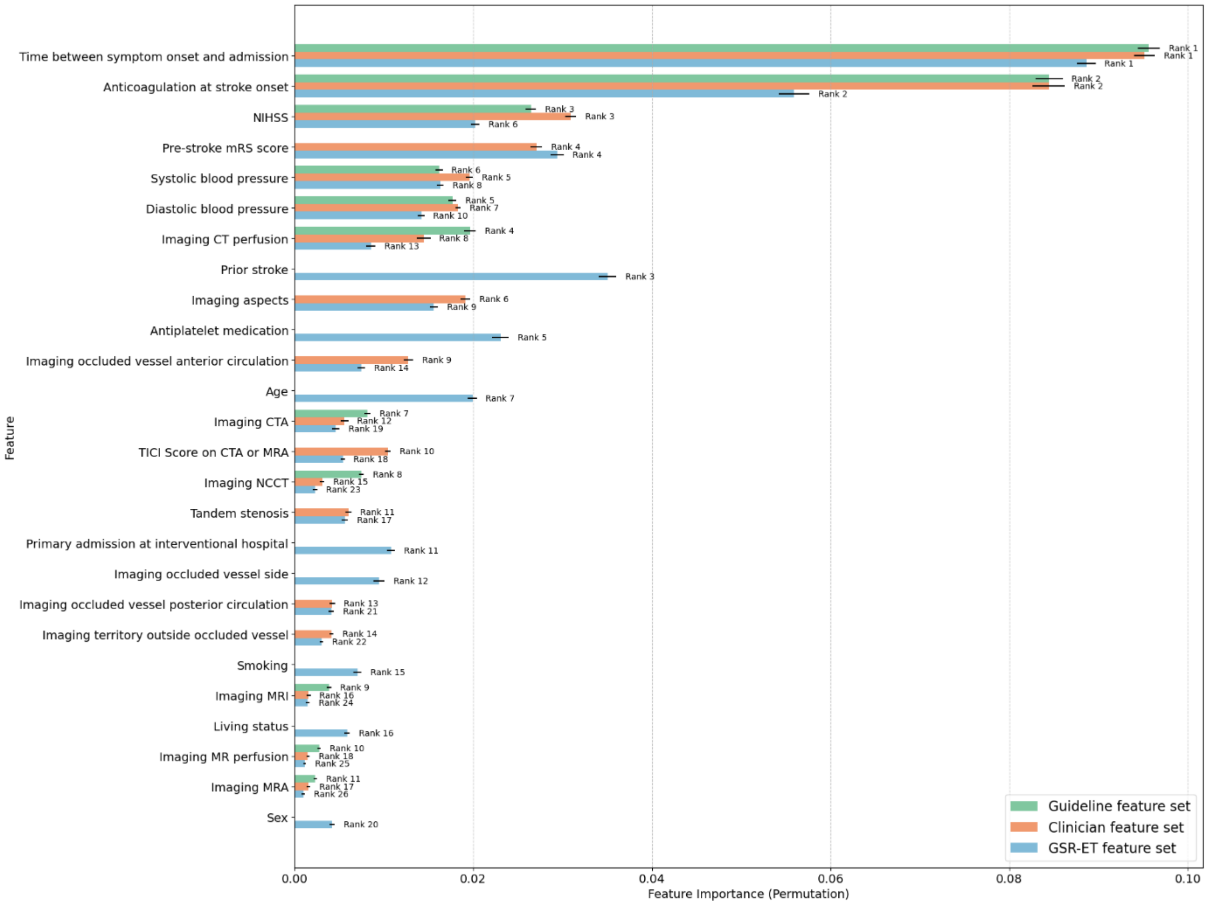

Supplement: Multimedia Appendix 5 [file medinform-v13-e69282-s005.pdf]
